# Molecular Modulation of Osteoblasts and Osteoclasts in Type 2 Diabetes

**DOI:** 10.1155/2018/6354787

**Published:** 2018-11-04

**Authors:** Selvalakshmi Rathinavelu, Crissy Guidry-Elizondo, Jameela Banu

**Affiliations:** ^1^Department of Health and Biomedical Sciences, College of Health Affairs, University of Texas Rio Grande Valley, 1201, W University Dr, Edinburg, TX 78539, USA; ^2^Department of Biology, College of Sciences, University of Texas Rio Grande Valley, 1201, W University Dr, Edinburg, TX 78539, USA

## Abstract

Diabetes is a common disease affecting majority of populations worldwide. Since 1980, there has been an increase in the number of people diagnosed as prediabetic and diabetic. Diabetes is characterized by high levels of circulating glucose and leads to most microvascular and macrovascular complications such as retinopathy, nephropathy, neuropathy, stroke, and myocardial infarction. Bone marrow vascular disruption and increased adiposity are also linked to various complications in type II diabetes mellitus. In addition to these complications, type 2 diabetic patients also have fragile bones caused by faulty mineralization mainly due to increased adiposity among diabetic patients that affects both osteoblast and osteoclast functions. Other factors that increase fracture risk in diabetic patients are increased oxidative stress, inflammation, and drugs administered to diabetic patients. This review reports the modulation of different pathways that affect bone metabolism in diabetic conditions.

## 1. Introduction

Diabetic patients are at high risk of developing osteoporosis. Normal to high bone mineral density (BMD) measurements recorded in type II diabetes mellitus (T2DM) patients are misleading [1]. In diabetic patients, an increase in the risk of hip (1.4–1.7-fold) and vertebral fractures have been reported [2]. As one ages, both genders are not only susceptible to increased risk of fragile bones but are also at high risk of developing diabetes, which augments the risk of bone fractures [3–6]. Bone fragility in T2DM patients is related to decreased bone strength and malformation of collagen fibers that can result in faulty mineralization and increased micro damages [7–9]. Using BMD measurements alone to diagnose bone condition in T2DM may not be reliable as the strength of the bone may be compromised in these patients. It is suggested that BMD with body mass index (BMI) adjustments may be a better indicator [10]. Supplemental data such as biochemical markers can be additional diagnostic tool. Bone biochemical markers such as C-terminal telopeptide (CTX) and N-terminal telopeptide (NTX) will reflect on the bone resorption process and breakdown of the collagen fibers. Interestingly, in T2DM patients, there is decreased CTX and increased NTX levels [11], and other reports did not find any difference between the two markers [12].

However, in T2DM patients, the quality of collagen fibers is compromised rather than increased breakdown of the collagen fibers. In T2DM patients, the trabecular bone network was shown to have large holes, decreased osteoblast recruitment, and mineral apposition rates combined with increased osteoclastogenesis [13].

The major pathophysiology in T2DM patients is insulin resistance (IR). This can be attributed to lack of or decreased insulin secretion and/or insulin receptors on the cell membranes. A close relationship between glucose and bone metabolism has been reported [14–18]. Yamaguchi and Sugimoto have described the link between glucose, fat, and bone metabolism [2]. They have suggested that osteocalcin, an important bone-forming marker, in the uncarboxylated form and the Wnt signalling pathway proteins, may be modulated to increase the fragility of bones in diabetic patients [19]. Other hormones secreted by adipocytes like adiponectin decrease IR [20], while leptin increases IR [21, 22]; moreover, advanced glycation end products (AGEs) and insulin-like growth factor-I (IGF-1), which regulate bones, may be also modified in T2DM [1, 2]. AGE is formed by elevated blood glucose levels that cause nonenzymatic glycosylation and binds to its receptor (RAGE) which activates transcription factor nuclear factor-*κ*B (NF-*κ*B). This results in increased expression of receptor activator of nuclear factor kappa-B ligand- (RANKL-) mediated osteoclastogenesis [23, 24]. Accumulation of AGE may also stimulate interleukins (IL) such as IL-6, which reduces osteoblast proliferation and activity while increasing osteoclastic activity [1, 25–29]. In T2DM patients, there is hypersecretion of calcium and decreased calcium absorption due to decreased vitamin D levels and estrogenic levels, especially in females [30].

In addition, drugs used to treat diabetes can also have an effect on bone health. One such group of drugs is thiazolidiones (TZD), which increases the risk of osteoporosis in T2DM patients. TZDs are capable of influencing the mesenchymal cells to differentiate more into adipocytes rather than osteoblasts which results in increased cortical porosity [31]. Furthermore, insulin is administered to diabetic patients to help lower circulating glucose which directly acts on osteoclasts. A review of the drug effects on bone can be found in Montagnani et al. [1]. Although metformin has been shown to reduce bone loss, based on the severity of the side effects caused by this drug [32], absorption of nutrients essential for bone health may be compromised [30].

## 2. Materials and Methods

In this review, we are presenting information on the interaction of different pathways that influence bone, glucose utilization, and insulin signalling pathways. We collected literature using the following search engines: PubMed, Google Scholar, Cochrane Reviews, and Medline. The keywords used for the search were type II diabetes, insulin, bone, insulin and osteoclasts, insulin and osteoblasts, insulin and Wnt, insulin and inflammation, and insulin and oxidative stress.

## 3. Results and Discussion

### 3.1. Insulin Signalling Pathway under Normal and Diabetic Conditions

**Figure 1:**
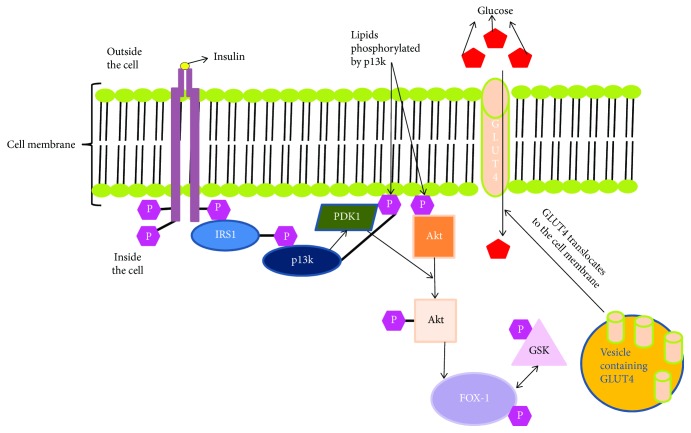
Insulin signalling pathway in normal cells.

Normally, insulin activates several cascades of intracellular signalling pathways, which begins with phosphorylation of insulin receptor substrate 1 & 2 (IRS-1 & 2) and is followed by activation of phosphotidylinositide 3 kinase (PI3-K) and protein kinase B (AKT). This series of phosphorylation events, in turn, deactivates forkhead box proteins (FOX) and phosphorylates glycogen synthase (GSK), which plays an important role in controlling gluconeogenesis, glycogenolysis, and maintaining glucose homeostasis [33] (Figure 1). *In vitro* DNA-binding assays and transfection experiments showed that both mammalian FoxO and FoxA proteins can bind to IRS and mediate transcriptional activation [34].

**Figure 2:**
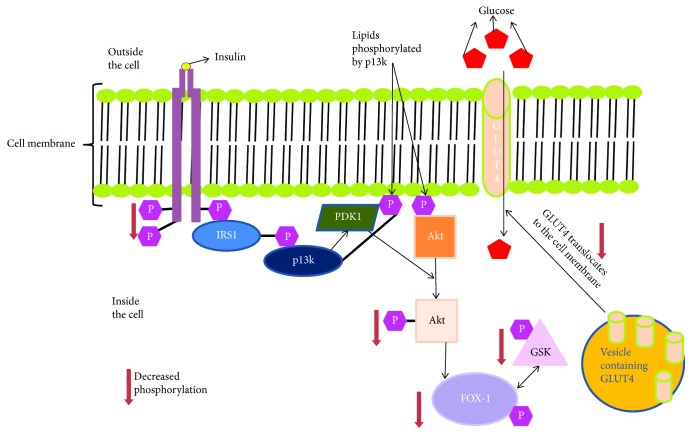
Insulin signalling pathway in cells of patients with type 2 diabetes mellitus.

Insulin regulates the transcriptional activity of hundreds of genes involved in glucose and lipid metabolism in the liver. Insulin along with growth hormone activates serine/threonine protein kinase (AKT), AKT phosphorylate FOXOs, and causes retention of FOXOs in the cytoplasm. In response to stress, decreased insulin, and growth hormone, FOXOs are activated and mediate bone cell functions [35, 36]. In T2DM, due to IR, there is decreased phosphorylation of IRS 1 & 2, decreasing PI3-K and increasing mitogen-activated protein kinase (MAPK) activation. This results in increased FOXO1 [33] (Figure 2). FOXO1 is activated in tissues associated with diabetic complications such as wound healing and bone fractures [33].

FOXOs play an important role in maintaining skeletal homeostasis by mediating both osteoclast and osteoblast function [35–41]. Other proteins like AGE, proinflammatory cytokines, and reactive oxygen species (ROS) are increased with high circulating blood glucose [33]. In T2DM, prolonged high levels of proinflammatory cytokines such as TNF-*α*, IL-1*β*, IL-6, and IL-18 enhance lipid peroxidation and dyslipidemia, resulting in increased osteoclastogenesis [42–44]. High levels of TNF-*α* increase the RANK/osteoprotegerin (OPG) ratio which enhances bone resorption [45].

Increased AGE, ROS, and proinflammatory cytokines increase bone loss. When AGE is formed, it bonds to its receptor RAGE and activates nuclear factor-*κ*B (NF-*κ*B) resulting in increased expression of RANKL-mediated osteoclastogenesis [23, 24, 46]. Prolonged inflammation also stimulates the expression of proapoptotic genes such as bcl-2-like protein (Bax). This reduces the expression of genes that stimulate osteoblast formation such as Fos-related antigen (FRA-1) and Runt-related transcription factor (RUNX2) [33] resulting in decreased bone formation. Oxidative stress reduces differentiation to osteoblasts and can directly degrade bone [47]. NF-*κ*B responds to oxidative stress and increases osteoclast activity and decreases osteoblast differentiation [47].

### 3.2. Type 2 Diabetes Modulation of Bone Marrow Stem Cells

The microenvironment in bone marrow cells is affected by complications of diabetes. The mesenchymal stem cells (MSC) can differentiate into adipocytes or osteoblasts depending on the prevailing signalling molecules. Long-standing diabetes causes disruption of the bone marrow microenvironment by depleting and altering stem/progenitor cells resulting in enhanced adipogenesis and depressed osteogenesis [3, 48–56]. *In vitro* studies on RAW264.7 cells have demonstrated that high glucose decreases autophagy of osteoclasts thereby increasing osteoclastogenesis [57]. The multifactorial causes of enhanced adipogenesis are augmented insulin signalling, hyperlipidemia, and ROS. One of the major players is peroxisome proliferator-activated receptor gamma (PPAR*γ*), an important regulator of lipid, glucose, and insulin metabolism. It consists of two isoforms—PPAR*γ*1 and PPAR*γ*2. PPAR*γ*2 regulates the differentiation of MSC to either adipocytes or osteoblast [58]. Inside the cells, high levels of blood glucose activate phosphatidylinositol 4,5 bisphosphate 3 kinase (PI3k) and phosphorylate protein kinase B (PKB). This decreases peroxisome proliferator-activated receptor gamma (PPAR*γ*) through FOXO1 and increases adipogenesis.

Activation of mechanistic target of rapamycin (mTOR) increases adipocyte specific factors in preadipocytes and increases muscle satellite cells [33]. The PI3K/PKB pathway is also stimulated by oxidative stress generated by ROS and enhances adipogenesis, thereby decreasing osteoclastogenesis [55].

### 3.3. FOXO1 Regulates RANKL-Mediated Osteoclastogenesis

FOX-1 is a transcription factor that mediates RANK-induced osteoclast formation. Osteoclast formation includes several steps such as differentiation of myeloid precursors to preosteoclasts, fusion of mononuclear preosteoclast to multinucleated osteoclasts, and maturation and activation of osteoclasts. Several proteins are involved in osteoclastogenesis such as RANKL, NK-*κ*B, TNF, macrophage colony stimulating factor (M-CSF), and nuclear factor of activated T cells (NFATC1) [36]. Initially, M-CSF binds to its receptor, which upregulates and activates RANK and NF-*κ*B in osteoclast precursor cells. RANK signalling stimulates the formation of single-cell tartrate-resistant acid phosphatase- (TRAP-) positive preosteoclasts, which fuse together to form the multinucleated TRAP-positive osteoclast [34].

Cell fusion is the most important step in osteoclastogenesis and dendritic cell-specific transmembrane protein (DC-STAMP), which induces NFATC1, the master gene in osteoclastogenesis [33]. Other NFATC1-mediated fusion molecules are TRAP, osteoclast-associated receptor (OSCAR), Cathepsin K, proto oncogene tyrosine protein kinase (C-SRC), and *β*3 integrin. Cell fusion can also be induced by other molecules independent of NFATc1 such as CD9, CD44, E-cadherin, merlin *α*, and macrophage fusion receptor [39]. When RANK stimulates NFATC1 through extracellular signal-regulated kinases (ERK)/c-Jun amino-terminal kinases (JNK)/MAP kinase p38 [33, 36], it also activates phospholipase C (PLC). This releases inositol triphosphate (IP_3_) which causes extracellular calcium influx and intracellular calcium release inducing calcium oscillation.

These calcium oscillations activated by NFATc1 are regulated by Transmembrane 64 (Tmem 64) and interact with sarcoplasmic endoplasmic reticulum carbonic anhydrase (SERCA), causing osteoclast differentiation [59]. Tmem64 also interacts with SERCA2 through tyrosine-based activation motif (ITAM) that has a common Fc receptor gamma subunit (FCR*γ*) and DNAX-activating protein 12 (DAP12). This costimulation leads to activation of phospholipase gamma (PLC*γ*) and IP_3_ causing calcium release from endoplasmic reticulum (ER), generating calcium oscillations. These oscillations activate Ca^2+^/calmodulin-dependent protein kinase (CaMK) IV and cyclic AMP response element-binding protein (CREB), which plays an important role in the generation of mitochondrial ROS, induction of NFATc1 and C-FOS necessary for osteoclast production. NFATc1 induced by CREB is short acting but continuously spike cycling Ca^2+^ by activating SERCA2, which is necessary to sustain NFATc1 activity during osteoclastogenesis [38]. Later, osteoclasts are polarized by actin, integrin *α*V, and integrin *β*3, which activates vacuolar ATPase and release of cathepsin K (CTSK), lysosomal cysteine, and protease to degrade bone matrix, causing bone resorption.

FOX-1 which mediates the effect of RANK on osteoclastogenesis also modulates Toll-like receptor (TLR) and cytokine production in monocyte and dendritic cells as well as downstream regulation of NFATC1. This, in turn, regulates dendritic cell-derived protein (DC-STAMP) and ATP 6VOD2, which play an important role in cell fusion [33]. The differentiated osteoclast expresses NFATc1, OSCAR, CTSK, and PPAR*γ*c1b. In diabetic conditions, increased RANKL/OPG ratio contributes to increased osteoclastogenesis [35]. Interestingly, in the absence of RANK, NF-*κ*B can be activated by TNF receptor-associated factor 6 (TRAF6) pathway, ectopic NFATC1, and activated RANKL promoters [41].

### 3.4. Role of FOXOs against Oxidative Stress in Osteoblasts

FOXOs protect osteoblasts from oxidative stress by interacting with transcription factor which regulates amino acid import, proliferation of osteoblasts, and generation of antioxidant enzymes such as catalase, superoxide dismutase (SOD), and glutathione [40]. In response to oxidative stress generated by reactive oxygen species (ROS), FOX 1, 3, and 4 are activated in the nucleus of the osteoblasts and produce antioxidants to decrease bone resorption. Oxidative stress is the critical step for osteoclast differentiation and function [35].

Both FOX1 and activating transcription factor 4 (ATF4) are located in the cytoplasm and respond to stress. Both are translocated to the nucleus which promotes protein and amino acid synthesis. ATF4 controls protein synthesis through a negative feedback mechanism that leads to accumulation of glutathione and collagen production. FOX1 also promotes osteoblast proliferation by increasing cell cycle cyclin D1 and D2 and suppressing cell cycle inhibitor p27Kip1. Decreased FOX1 suppresses osteoblastogenesis by decreasing osterix and type 1 collagen protein levels but does not affect levels of Runx2 and Bsp (bone sialoprotein) [40]. FOXOs are potent repressors of osteoblastogenesis by also decreasing PPAR-*γ* [37, 40]. This increase in bone resorption may be attributed to activation of antiosteoclastogenic factor osteoprotegerin (OPG) which promotes FOX-mediated transcription of *β*-catenin/T-cell specific transcription factor (TCF), thereby promoting RANK-mediated osteoclastogenesis by increasing PPAR-*γ*. This increases apoptosis of osteocytes and enhances adipogenesis as indicated by decreased bone markers such as calcitonin, TRAP, and cathepsin K [37]. Osteoblasts exposed to oxidative stress also have decreased expression of RUNX2 and osteocalcin and increased adipogenesis-related factors PPAR-*γ* and fatty acid binding protein-4 (FABP4) [55].

### 3.5. Wnt/*β*-Catenin Pathways in Metabolic Syndrome

Activation of Wnt (*β*-catenin) signalling promotes differentiation of progenitor stem cells into osteoblasts and prevents adipogenesis. Regulation of Wnt signalling is a balance between adipogenesis and myogenesis [60]. Wnt/(*β*-catenin) is activated when PPAR-*γ* binds with lymphoid enhancer factor/T cell factor (LEF/TCF), binding domain of *β*-catenin, and facilitates its phosphorylation by glycogen synthase kinase3b (GSK3b), thereby resulting in increased differentiation within preadipocytes [61, 62]. The Wnt family has 19 ligands, 10 Wnt receptors, frizzled (Fz) coreceptors, and low-density lipoprotein receptor-related proteins (LRP-5 and LRP-6). The actions of Wnt include canonical and noncanonical pathways. Noncanonical Wnt signalling cascade also plays an important role in adipogenesis. Wnt binds to its receptor and activates phospholipase C (PLC), generating diacylglycerol (DAG) and inositol triphosphate (IP_3_), which results in the release of intracellular calcium from the endoplasmic reticulum. Efflux of intracellular calcium activates protein kinase C (PKC). This leads to the phosphorylation of SET domain bifurcated-1 (SETB1) histone methyltransferase, inhibiting PPAR-*γ* through histone methylation H3-K9, and upregulates RUNX2 required for osteoblastogenesis [63, 64]. PKC has a dual role in adipogenesis. Its isoforms *α*, *δ*, and *μ* inhibit adipogenesis, while *β*1 and *γ* isoforms promote adipogenesis. Hyperglycemia-induced noncanonical Wnt pathway increased adipogenesis through activation of various PKC isoforms [55]. Wnt/*β* catenin pathway inhibitor, sclerostin, is increased in the serum of T2DM and is inversely related to levels of bone turnover markers [65, 66].

Regulation of Wnt signalling is a balance between adipogenesis and myogenesis. Insulin action and insulin resistance can be modulated by Wnt and lipoprotein receptor-related protein 5 (LRP5) activity [57]. Wnt canonical pathway acts on Fz/(LRP5/6), decreasing GSK-3*β* and increasing *β*-catenin, which translocates to the nucleus, conjuncts with lymphoid enhancer factor/T-cell factor (TCF), and regulates transcription of Wnt target genes. The *in vitro* study using human adipose-derived stem cells has shown that during the differentiation of insulin-producing cells (IPC), protein levels of Wnt 1, *β*-catenin, and GSK3*β* are increased [67]. At the same time, TCF-1 and cyclin-D increased from day 1 to day 9 and decreased from day 9 onwards and continued to decrease. Inhibition of Wnt signalling does not decrease differentiation from day 1 to day 9 but upregulates IPC specific markers such as insulin promoter 1 (PDX-1), insulin, and insulin receptor substrates 1 and 2 (IRS-1 and 2) from day 9 to day 12. Wnt signalling specific marker such as glucokinase decreased from day 9 to day 12. Activation of Wnt signalling on day 9 decreases IPC specific markers, and deactivation of Wnt signalling is necessary for IPC maturation [39, 67]. Overall, Wnt signalling may be more involved in IPC maturation [58]. In the pancreas and hepatocytes, *β* catenin/Wnt signalling pathways activate glucokinase promoter activities in the presence of PPAR-*γ* and cyclin-D promoter with transcription factor 7-like 2 (TCF7L2) binding sites [68] and play an important role in maintaining *β*-cell function [69].

IRS-2 and Akt are key signalling molecules in maintaining *β*-cell mass [70]. Akt prevents free fatty acid-induced *β*-cell apoptosis through inhibition of proapoptotic proteins like germinal center kinase 3*α*, *β*(GCK3*α*/*β*), FOX 1, and p53 [70]. The cross talk between insulin and Wnt signalling occurs at the level of coreceptor LRP 5, which has a profound positive effect on insulin signalling in preadipocytes [71].

The direct interaction between insulin receptors and LRP 5 occurs in an insulin/Wnt inducible manner. Insulin receptor/LRP 5 plays an important role in the pathogenesis of IR and obesity. Decreased Wnt canonical pathway receptor LRP5/6 increases the risk of diabetes mellitus and impaired glucose intolerance [33, 60].

Wnt signalling varies slightly in different cells. In preadipocytes, both insulin and Wnt3a lead to phosphorylation of LRP 6, GSK3b, Akt, and extracellular signal-regulated kinase (ERK1/2). If both IGF receptors and insulin receptors decrease, insulin-mediated Wnt3a phosphorylation decreases. Whereas, Wnt-mediated phosphorylation decreases not only when insulin receptors and IGF receptors are decreased but also in the absence of these receptors.

In skeletal muscles, Wnt/*β*-catenin signalling (1) increases muscle-specific myogenic transcription factor, (2) decreases PPAR-*γ*-related adipogenesis and C/EBP *α* expression, (3) converts type 2 skeletal muscle fibers into type 1 muscle fibers, (4) decreases c-myc-mediated activation of p27, which decreases myogenesis and increases adipogenesis, and (5) activates mitogenic factor 5 (Myf 5), which in turn activates myoblast determination protein D (myoD) [72, 73]. Risk of T2DM increases with decreased Wnt signalling in the skeletal muscle and increased adipogenesis [55, 74].

In hepatocytes, canonical Wnt3a stimulation decreases key enzymes of gluconeogenesis such as phosphoenolpyruvate carboxykinase (PEPCK) and glucose 6-phosphatase (G6Pase). Noncanonical Wnt 11 stimulation decreases glucose output by hepatocytes. Insulin increases TCF7L2, which increases cyclin D, a downstream target of the Wnt signalling pathway [75]. *β*-Catenin phosphorylation is positively correlated with transcriptional activity of *β*-cat/TCF. *β*-catenin phosphorylation occurs with (1) protein kinase A (PKA) activation; (2) PKA/cyclic adenosine monophosphate (cAMP) activator and glucagon stimulate cAMP responsive element-binding protein (CREB) phosphorylation, and (3) insulin is able to stimulate *β*-catenin phosphorylation [76].

In response to feeding, insulin mediates a repressor effect on gluconeogenesis through TCF7L2 and *β*-catenin phosphorylation. In the absence of TCF7L2, insulin also decreases gluconeogenesis by attenuating FOX [77]. Wnt and TCF7L2 are negative regulators of gluconeogenesis, while FOX is a positive regulator of gluconeogenesis [76]; insulin increases TCF7L2 in intestinal L-cells and stimulates the expression of proglucagon gene and incretin hormone glucagon-like peptide 1 (GLP-1) [77]; glucagon increases FOX through cAMP, and insulin decreases FOX through PI3K/Akt-mediated nuclear exclusion of FOXO1 [78], and TCF7L2 increases hepatic glucose production and the risk of T2DM [77].

### 3.6. Hormonal Balance in Skeletal Homeostasis and Metabolic Syndrome

Certain hormones associated with bone metabolism and energy balance such as osteocalcin, leptin, and adiponectin affect insulin signalling pathways and other hormones related to calcium homeostasis [70]. Osteocalcin is the marker of osteoblast activity [79] and is modulated in the osteoblast-specific gene esp, which encodes osteotesticular protein tyrosine phosphatase (OST-PTP) [80]. This OST-PTP dephosphorylates the insulin receptor [79]. Decreased OST-PTP increases insulin signalling through the insulin receptor and promotes pancreatic *β*-cell proliferation, while increasing insulin and insulin-sensitizing adiponectin [79, 81].

Leptin produced by adipocytes acts on the hypothalamus, which regulates decreases and increases on appetite and satiety, as well as decrease in bone formation by inhibiting osteocalcin production in new bones [82, 83]. Adiponectin, also produced by adipocytes, acts on bones in an age-dependent manner and is inversely proportional to BMD [84]. Adiponectin is an antagonist to leptin, acts on the brain, and increases sympathetic output to peripheral osteoblast [85].

Other hormones like vitamin D and estrogen levels may be associated with developing T2DM and IR [65, 86]. Decreased levels of vitamin D decrease renal reabsorption of calcium and reduce osteocalcin production by osteoblasts [87], resulting in diminished bone formation. Estrogen, on the other hand, is implicated in IR. Of the two estrogen receptors, ER*α* is positively associated with glucose metabolism [88]. Both direct and indirect effects of ER*α* on IR have been reported. Directly, ER*α* can act on insulin signalling and increase GLUT4 expression, while indirectly, it can modulate oxidative stress and inflammation [89].

### 3.7. Influence of Drugs Used for Treating Diabetes and Bone

Different drugs commonly prescribed for treating diabetes like metformin, thiazolidinedione, and sulfonylurea do affect bone mass (Table 1). Most of them act on different pathways to influence bone status in the patients. One highly prescribed medication for T2DM patients is metformin. This drug has been associated with the most bone protective properties. Metformin can activate adenosine monophosphate kinase (AMPK) to reduce indigenous glucose production or may also act independent of the AMPK pathway by inhibiting glycolytic enzymes or adenylate cyclase and decreases gluconeogenesis [90, 91]. At the mesenchymal cellular level, metformin reduces adipocyte formation in the bone marrow by preventing endothelial nitric oxide synthase (eNOS) expression [14]. Metformin acts as an insulin sensitizer, increases GLP-1 secretion in L-cells of the intestines, stimulates nuclear translocation of *β*-catenin, and increases transcription of luciferase reporter gene. GLP-1 increases (1) pancreatic insulin secretion, (2) proinsulin gene expression, and (3) *β*-cell mass. GLP-1 also decreases gastric emptying and glucagon release. Inside the cells, metformin increases IRS-2, p-PI3K, p-PKB, calcium/calmodulin-dependent protein kinase 2 (CaMK2), CREB, p-GSKS*β* (inactive form), enzymes of glycolysis like phosphofructokinase (PFK), and Kreb's cycle enzymes (isocitrate dehydrogenase, malate dehydrogenase). Glucose utilization mediated by metformin is through calcium-dependent protein kinase [55]. Metformin also increases the markers of osteogenic differentiation and function [33]. Although metformin has beneficial effects on bone, there is concern for patients who have moderate to severe digestive intolerance after consuming this medication, as nutrients necessary for bone health may not be absorbed properly.

Sulfonylureas are organic compounds that act on the pancreatic cells and increase the release of insulin. They act on membrane channels, first by blocking the potassium channels, causing depolarization in the cell which then opens the Ca^2+^ channels and this increases the release of insulin [92]. There are very few studies that show the interaction between the consumption of these drugs and bone. The few reports show that C-terminal telopeptide (CTX) and N-terminal telopeptide (NTX) levels are decreased in patients who take these drugs [32, 93]. Sulfonylureas can also activate the PI3K/ATK pathway which then increases the expression of alkaline phosphatase and osteocalcin mRNA expression [94]. It has been reported that sulfonylureas protect against ovariectomy-induced bone loss and also increase the mechanical strength by increasing bone formation [95]. Based on the limited evidence available, sulfonylureas are beneficial to the bone. In-depth, long-term studies are necessary to know the exact function of sulfonylureas on the bone in T2DM patients.

Recently, incretin-based therapies are being used. Incretins are inhibitors of glucagon-like peptide 1 receptors (GLP-1) and dipeptidyl peptidase 4 (DPP-4). GLP-1 receptors are expressed in the pancreatic *β* cells and other cells promoting metabolic activity [32]. It has been reported that GLP-1 can control bone resorption by interacting with GLP-2 and glucose-dependent insulinotropic polypeptide (GIP) [96]. In addition, it can also act on a calcitonin-dependent pathway [96]. GLP-2 may have antiresorptive function [97] while GIP can influence bone resorption and bone formation [80, 98].

Many clinical studies with patients who are on insulin treatment for T2DM have reported increased risk of fracture, especially in postmenopausal women [99–101]. Insulin is known to increase bone formation generally, and lack of detailed study on the reasons for insulin increasing fracture rates is not understood.

Amylin is a peptide that is reported to have some effects on bone metabolism [32]. Both *in vitro* and *in vivo* studies show this peptide, when present in lower levels, is associated with inhibited osteoclastic activity [102]. However, there is not much data on clinical studies regarding the effects of amylin on the bone.

Sodium glucose cotransporter inhibitors (SGCT) are drugs that reduce the reabsorption of glucose from the kidneys by inhibiting the sodium glucose cotransporters. They are capable of altering the calcium and phosphate homeostasis; therefore, they affect the bone and may be more deleterious to the bone. Several reports have shown that imbalance in the calcium and phosphate homeostasis triggers secretion of PTH which increases bone resorption [103]. Increased levels of CTX and decreased BMD values are also reported with the use of these drugs [104]. However, some reports have not shown any significant influence on the mineral levels or the levels of parathyroid hormone (PTH) and vitamin D [105, 106]. This may be due to any differences in the intake of vitamins and minerals. At this point, it may be safe to say that these drugs may be deleterious to the bone although more in-depth studies are required to determine the mechanism by which these drugs affect the bone [32].

TZDs are the most popular set of drugs that are proven to be harmful to the bone. The primary mechanism of action in TZDs is through direct induction of PPAR-*γ* leading to improved insulin sensitivity. The stimulation of differentiation of multipotent stem cells into adipocytes and increased adiposity in bone marrow are seen in patients treated with TZDs. The most common side effect of TZDs is weight gain through promotion of PPAR-*γ*, with increased adipogenesis leading to increased subcutaneous and bone marrow fat depots and decreased bone formation. These properties of TZDs make the patients' bones become very fragile, and their BMD is significantly decreased [33].

## 4. Conclusion

There is increasing evidence about the interaction between the glucose metabolic pathway, insulin signalling, and bone metabolic pathways. In 2015, it was reported that there was a rise in people aged 66 and older having T2DM [107]. Unfortunately, this is also the age when both men and women have decreased bone mass and are at high risk of having hip and spine fractures [65]. This may be because of the interaction of signalling pathways that modulate bone and glucose metabolism in T2DM patients. In order to assess the fracture risk, a combination of BMD, FRAX, and biochemical markers should be used. T2DM patients should be tested for their bone health regularly, and bone status in T2DM patients should be recognized as a complication of diabetes as recommended by Sanches et al. [58]. Another factor that influences the bone in T2DM is as a side effect of the drugs that are prescribed to treat diabetes. It is important to note that there is an intricate connection between the different pathways that are altered in T2DM patients and bone metabolism. Although there is evidence of the effects of metformin and TZDs on bones, more research need to be conducted with the newer antidiabetic drugs. Therefore, patients being treated for diabetes should be tested for several vitamin and mineral levels. This information should be used to advise patients on the nutrient intake of specific vitamin and mineral deficiencies. In addition, medications used for treating diabetes should be carefully chosen, and any micronutrient deficiency should be supplemented.

## Figures and Tables

**Table 1:** Effects of antidiabetic drugs on bone metabolism.

Antidiabetic drugs	Mode of action	References
Amylin	At low concentrations ⬇ osteoclastogenesis	[90]
Incretin	⬇ GLP-1 receptors; ⬆ GIP influence on bone resorption and bone formation	[30, 71, 86]
Insulin	⬆ Bone formation	[87–89]
Metformin	⬇ Indigenous glucose production; ⬆ insulin sensitivity; ⬆ osteogenic markers	[31, 51, 78, 79, 80–82]
Sodium glucose cotransporter inhibitors	Interferes with calcium and phosphate homeostasis; ⬆ CTX and ⬇ BMD	[91, 92]
Sulfonylureas	⬇ CTX, NTX; ⬆ ALP, osteocalcin, bone strength	[30, 81, 83]
Thiazolidinediones	⬆ Adipogenesis; ⬇ BMD	[31]

⬆ = increases; ⬇ = decreases. GLP-1 = Glucogon like peptide 1, GIP = G;ucose-dependent insulinotropic polypeptide, CTX = C-terminal telopeptide, NTX = N-terminal telopeptide, BMD = bone mineral density, ALP = alkaline phosphatase.

## References

[1] Montagnani A., Gonnelli S., Alessandri M., Nuti R. (2011). Osteoporosis and risk of fracture in patients with diabetes: an update. *Aging Clinical and Experimental Research*.

[2] Yamaguchi T., Sugimoto T. (2011). Bone metabolism and fracture risk in type 2 diabetes mellitus [review]. *Endocrine Journal*.

[3] Janghorbani M., Van Dam R. M., Willett W. C., Hu F. B. (2007). Systematic review of type 1 and type 2 diabetes mellitus and risk of fracture. *American Journal of Epidemiology*.

[4] Petit M. A., Paudel M. L., Taylor B. C. (2009). Bone mass and strength in older men with type 2 diabetes: the Osteoporotic Fractures in Men Study. *Journal of Bone and Mineral Research*.

[5] Khoo C. L., Perera M. (2005). Diabetes and the menopause. *The Journal of the British Menopause Society*.

[6] Merlotti D., Gennari L., Dotta F., Lauro D., Nuti R. (2010). Mechanisms of impaired bone strength in type 1 and 2 diabetes. *Nutrition, Metabolism, and Cardiovascular Diseases*.

[7] Dixit P. K., Ekstrom R. A. (1980). Decreased breaking strength of diabetic rat bone and its improvement by insulin treatment. *Calcified Tissue International*.

[8] Saito M., Marumo K. (2009). Collagen cross-links as a determinant of bone quality: a possible explanation for bone fragility in aging, osteoporosis, and diabetes mellitus. *Osteoporosis International*.

[9] Reddy G. K., Stehno-Bittel L., Hamade S., Enwemeka C. S. (2001). The biomechanical integrity of bone in experimental diabetes. *Diabetes Research and Clinical Practice*.

[10] Bowden D. W., Cox A. J., Freedman B. I. (2010). Review of the Diabetes Heart Study (DHS) family of studies: a comprehensively examined sample for genetic and epidemiological studies of type 2 diabetes and its complications. *The Review of Diabetic Studies*.

[11] Starup-Linde J., Eriksen S. A., Lykkeboe S., Handberg A., Vestergaard P. (2014). Biochemical markers of bone turnover in diabetes patients--a meta-analysis, and a methodological study on the effects of glucose on bone markers. *Osteoporosis International*.

[12] Herrmann M., Seibel M. J. (2008). The amino- and carboxyterminal cross-linked telopeptides of collagen type I, NTX-I and CTX-I: a comparative review. *Clinica Chimica Acta*.

[13] Pritchard J. M., Giangregorio L. M., Atkinson S. A. (2011). Association of larger holes in the trabecular bone at the distal radius in postmenopausal women with type 2 diabetes mellitus compared to controls. *Arthritis Care & Research*.

[14] Gu Q., Gu Y., Yang H., Shi Q. (2017). Metformin enhances osteogenesis and suppresses adipogenesis of human chorionic villous mesenchymal stem cells. *The Tohoku Journal of Experimental Medicine*.

[15] Starup-Linde J., Vestergaard P. (2016). Biochemical bone turnover markers in diabetes mellitus - a systematic review. *Bone*.

[16] Starup-Linde J., Frost M., Vestergaard P., Abrahamsen B. (2016). Epidemiology of fractures in diabetes. *Calcified Tissue International*.

[17] Starup-Linde J., Gregersen S., Vestergaard P. (2016). Associations with fracture in patients with diabetes: a nested case-control study. *BMJ Open*.

[18] Starup-Linde J., Lykkeboe S., Gregersen S. (2016). Differences in biochemical bone markers by diabetes type and the impact of glucose. *Bone*.

[19] Motyl K. J., McCabe L. R., Schwartz A. V. (2010). Bone and glucose metabolism: a two-way street. *Archives of Biochemistry and Biophysics*.

[20] Confavreux C. B., Levine R. L., Karsenty G. (2009). A paradigm of integrative physiology, the crosstalk between bone and energy metabolisms. *Molecular and Cellular Endocrinology*.

[21] de Paula F. J. A., Horowitz M. C., Rosen C. J. (2010). Novel insights into the relationship between diabetes and osteoporosis. *Diabetes/Metabolism Research and Reviews*.

[22] Wolf G. (2008). Energy regulation by the skeleton. *Nutrition Reviews*.

[23] Ding K. H., Wang Z. Z., Hamrick M. W. (2006). Disordered osteoclast formation in RAGE-deficient mouse establishes an essential role for RAGE in diabetes related bone loss. *Biochemical and Biophysical Research Communications*.

[24] Xie J., Mendez J. D., Mendez-Valenzuela V., Aguilar-Hernandez M. M. (2013). Cellular signalling of the receptor for advanced glycation end products (RAGE). *Cellular Signalling*.

[25] Hernandez C. J., Tang S. Y., Baumbach B. M. (2005). Trabecular microfracture and the influence of pyridinium and non-enzymatic glycation-mediated collagen cross-links. *Bone*.

[26] Katayama Y., Akatsu T., Yamamoto M., Kugai N., Nagata N. (1996). Role of nonenzymatic glycosylation of type I collagen in diabetic osteopenia. *Journal of Bone and Mineral Research*.

[27] Miyata T., Notoya K., Yoshida K. (1997). Advanced glycation end products enhance osteoclast-induced bone resorption in cultured mouse unfractionated bone cells and in rats implanted subcutaneously with devitalized bone particles. *Journal of the American Society of Nephrology*.

[28] Takagi M., Kasayama S., Yamamoto T. (1997). Advanced glycation endproducts stimulate interleukin-6 production by human bone-derived cells. *Journal of Bone and Mineral Research*.

[29] Wang X., Shen X., Li X., Mauli Agrawal C. (2002). Age-related changes in the collagen network and toughness of bone. *Bone*.

[30] Isaia G., Bodrato L., Carlevatto V., Mussetta M., Salamano G., Molinatti G. M. (1987). Osteoporosis in type II diabetes. *Acta Diabetologica Latina*.

[31] Sardone L. D., Renlund R., Willett T. L., Fantus I. G., Grynpas M. D. (2011). Effect of rosiglitazone on bone quality in a rat model of insulin resistance and osteoporosis. *Diabetes*.

[32] Chandran M. (2017). Diabetes drug effects on the skeleton. *Calcified Tissue International*.

[33] Jiao H., Xiao E., Graves D. T. (2015). Diabetes and its effect on bone and fracture healing. *Current Osteoporosis Reports*.

[34] Hannenhalli S., Kaestner K. H. (2009). The evolution of Fox genes and their role in development and disease. *Nature Reviews. Genetics*.

[35] Bartell S. M., Kim H. N., Ambrogini E. (2014). FoxO proteins restrain osteoclastogenesis and bone resorption by attenuating H_2_O_2_ accumulation. *Nature Communications*.

[36] Wang Y., Dong G., Jeon H. H. (2015). FOXO1 mediates RANKL-induced osteoclast formation and activity. *Journal of Immunology*.

[37] Ambrogini E., Almeida M., Martin-Millan M. (2010). FoxO-mediated defense against oxidative stress in osteoblasts is indispensable for skeletal homeostasis in mice. *Cell Metabolism*.

[38] Kim H., Kim T., Jeong B. C. (2013). Tmem64 modulates calcium signaling during RANKL-mediated osteoclast differentiation. *Cell Metabolism*.

[39] Kim K., Lee S. H., Ha Kim J., Choi Y., Kim N. (2008). NFATc1 induces osteoclast fusion via up-regulation of Atp6v0d2 and the dendritic cell-specific transmembrane protein (DC-STAMP). *Molecular Endocrinology*.

[40] Rached M. T., Kode A., Xu L. (2010). FoxO1 is a positive regulator of bone formation by favoring protein synthesis and resistance to oxidative stress in osteoblasts. *Cell Metabolism*.

[41] Takayanagi H., Kim S., Koga T. (2002). Induction and activation of the transcription factor NFATc1 (NFAT2) integrate RANKL signaling in terminal differentiation of osteoclasts. *Developmental Cell*.

[42] Graves D. T., Kayal R. A. (2008). Diabetic complications and dysregulated innate immunity. *Frontiers in Bioscience*.

[43] Moseley K. F. (2012). Type 2 diabetes and bone fractures. *Current Opinion in Endocrinology, Diabetes, and Obesity*.

[44] Yamagishi S. (2011). Role of advanced glycation end products (AGEs) in osteoporosis in diabetes. *Current Drug Targets*.

[45] Pacios S., Kang J., Galicia J. (2012). Diabetes aggravates periodontitis by limiting repair through enhanced inflammation. *The FASEB Journal*.

[46] Makita Z., Radoff S., Rayfield E. J. (1991). Advanced glycosylation end products in patients with diabetic nephropathy. *The New England Journal of Medicine*.

[47] Abdollahi M., Larijani B., Rahimi R., Salari P. (2005). Role of oxidative stress in osteoporosis. *Therapy*.

[48] Botolin S., Faugere M. C., Malluche H., Orth M., Meyer R., McCabe L. R. (2005). Increased bone adiposity and peroxisomal proliferator-activated receptor-gamma2 expression in type I diabetic mice. *Endocrinology*.

[49] Botolin S., McCabe L. R. (2007). Bone loss and increased bone adiposity in spontaneous and pharmacologically induced diabetic mice. *Endocrinology*.

[50] de Liefde I. I., van der Klift M., de Laet C. E. D. H., van Daele P. L. A., Hofman A., Pols H. A. P. (2005). Bone mineral density and fracture risk in type-2 diabetes mellitus: the Rotterdam Study. *Osteoporosis International*.

[51] Dhaliwal R., Cibula D., Ghosh C., Weinstock R. S., Moses A. M. (2014). Bone quality assessment in type 2 diabetes mellitus. *Osteoporosis International*.

[52] Janghorbani M., Feskanich D., Willett W. C., Hu F. (2006). Prospective study of diabetes and risk of hip fracture: the Nurses’ Health Study. *Diabetes Care*.

[53] Leidig-Bruckner G., Ziegler R. (2001). Diabetes mellitus a risk for osteoporosis?. *Experimental and Clinical Endocrinology & Diabetes*.

[54] Oikawa A., Siragusa M., Quaini F. (2010). Diabetes mellitus induces bone marrow microangiopathy. *Arteriosclerosis, Thrombosis, and Vascular Biology*.

[55] Piccinin M. A., Khan Z. A. (2014). Pathophysiological role of enhanced bone marrow adipogenesis in diabetic complications. *Adipocytes*.

[56] Yaturu S., Bryant B., Jain S. K. (2007). Thiazolidinedione treatment decreases bone mineral density in type 2 diabetic men. *Diabetes Care*.

[57] Cai Z. Y., Yang B., Shi Y. X. (2018). High glucose downregulates the effects of autophagy on osteoclastogenesis via the AMPK/mTOR/ULK1 pathway. *Biochemical and Biophysical Research Communications*.

[58] Sanches C. P., Vianna A. G. D., Barreto F. d. C. (2017). The impact of type 2 diabetes on bone metabolism. *Diabetology and Metabolic Syndrome*.

[59] Arvanitis D. A., Vafiadaki E., Fan G. C. (2007). Histidine-rich Ca-binding protein interacts with sarcoplasmic reticulum Ca-ATPase. *American Journal of Physiology. Heart and Circulatory Physiology*.

[60] Palsgaard J., Emanuelli B., Winnay J. N., Sumara G., Karsenty G., Kahn C. R. (2012). Cross-talk between insulin and Wnt signaling in preadipocytes: role of Wnt co-receptor low density lipoprotein receptor-related protein-5 (LRP5). *The Journal of Biological Chemistry*.

[61] Farmer S. R. (2005). Regulation of PPAR*γ* activity during adipogenesis. *International Journal of Obesity*.

[62] Miller J. R. (2002). The Wnts. *Genome Biology*.

[63] Fleming I., MacKenzie S. J., Vernon R. G., Anderson N. G., Houslay M. D., Kilgour E. (1998). Protein kinase C isoforms play differential roles in the regulation of adipocyte differentiation. *The Biochemical Journal*.

[64] Zhou Y., Wang D., Li F., Shi J., Song J. (2006). Different roles of protein kinase C-*β*I and -*δ* in the regulation of adipocyte differentiation. *The International Journal of Biochemistry & Cell Biology*.

[65] Rubin M. R. (2015). Bone cells and bone turnover in diabetes mellitus. *Current Osteoporosis Reports*.

[66] Canalis E. (2013). Wnt signalling in osteoporosis: mechanisms and novel therapeutic approaches. *Nature Reviews Endocrinology*.

[67] Shi Q., Luo S., Jia H. (2013). Wnt/*β*-catenin signaling may be involved with the maturation, but not the differentiation, of insulin-producing cells. *Biomedicine & Pharmacotherapy*.

[68] Welters H. J., Kulkarni R. N. (2008). Wnt signaling: relevance to *β*-cell biology and diabetes. *Trends in Endocrinology and Metabolism*.

[69] Mei J., Holst L. S., Landstrom T. R. (2002). C_2_-ceramide influences the expression and insulin-mediated regulation of cyclic nucleotide phosphodiesterase 3B and lipolysis in 3T3-L1 adipocytes. *Diabetes*.

[70] Kim M. H., Hong S. H., Lee M. K. (2013). Insulin receptor-overexpressing *β*-cells ameliorate hyperglycemia in diabetic rats through Wnt signaling activation. *PLoS One*.

[71] Cselenyi C. S., Jernigan K. K., Tahinci E., Thorne C. A., Lee L. A., Lee E. (2008). LRP6 transduces a canonical Wnt signal independently of Axin degradation by inhibiting GSK3’s phosphorylation of *β*-catenin. *Proceedings of the National Academy of Sciences of the United States of America*.

[72] Armstrong D. D., Esser K. A. (2005). Wnt/*β*-catenin signaling activates growth-control genes during overload-induced skeletal muscle hypertrophy. *American Journal of Physiology. Cell Physiology*.

[73] Tajbakhsh S., Borello U., Vivarelli E. (1998). Differential activation of Myf5 and MyoD by different Wnts in explants of mouse paraxial mesoderm and the later activation of myogenesis in the absence of Myf5. *Development*.

[74] Zhou D., Strakovsky R. S., Zhang X., Pan Y. X. (2012). The skeletal muscle Wnt pathway may modulate insulin resistance and muscle development in a diet-induced obese rat model. *Obesity*.

[75] Lyssenko V., Lupi R., Marchetti P. (2007). Mechanisms by which common variants in the *TCF7L2* gene increase risk of type 2 diabetes. *The Journal of Clinical Investigation*.

[76] Smith P. W., Rusnak P. G. (1991). APIC guideline for infection prevention and control in the long-term care facility. *American Journal of Infection Control*.

[77] Ip W., Shao W., Chiang Y. T. A., Jin T. (2012). The Wnt signaling pathway effector TCF7L2 is upregulated by insulin and represses hepatic gluconeogenesis. *American Journal of Physiology. Endocrinology and Metabolism*.

[78] Wegner L., Hussain M. S., Pilgaard K. (2008). Impact of *TCF7L2* rs7903146 on insulin secretion and action in young and elderly Danish twins. *The Journal of Clinical Endocrinology and Metabolism*.

[79] Zhang Q., Riddle R. C., Clemens T. L. (2015). Bone and the regulation of global energy balance. *Journal of Internal Medicine*.

[80] Zhong Q., Itokawa T., Sridhar S. (2007). Effects of glucose-dependent insulinotropic peptide on osteoclast function. *American Journal of Physiology. Endocrinology and Metabolism*.

[81] Ferron M., Wei J., Yoshizawa T. (2010). Insulin signaling in osteoblasts integrates bone remodeling and energy metabolism. *Cell*.

[82] Ducy P., Amling M., Takeda S. (2000). Leptin inhibits bone formation through a hypothalamic relay: a central control of bone mass. *Cell*.

[83] Takeda S., Elefteriou F., Levasseur R. (2002). Leptin regulates bone formation via the sympathetic nervous system. *Cell*.

[84] Wiklund P. K., Xu L., Wang Q. (2012). Lactation is associated with greater maternal bone size and bone strength later in life. *Osteoporosis International*.

[85] Richards J. B., Valdes A. M., Burling K., Perks U. C., Spector T. D. (2007). Serum adiponectin and bone mineral density in women. *The Journal of Clinical Endocrinology and Metabolism*.

[86] Mathieu C., Gysemans C., Giulietti A., Bouillon R. (2005). Vitamin D and diabetes. *Diabetologia*.

[87] Chaiban J. T., Nicolas K. G. (2015). Diabetes and bone still a lot to learn. *Clinical Reviews in Bone and Mineral Metabolism*.

[88] Zirilli L., Rochira V., Diazzi C., Caffagni G., Carani C. (2008). Human models of aromatase deficiency. *The Journal of Steroid Biochemistry and Molecular Biology*.

[89] Gupte A. A., Pownall H. J., Hamilton D. J. (2015). Estrogen: an emerging regulator of insulin action and mitochondrial function. *Journal of Diabetes Research*.

[90] Madiraju A. K., Erion D. M., Rahimi Y. (2014). Metformin suppresses gluconeogenesis by inhibiting mitochondrial glycerophosphate dehydrogenase. *Nature*.

[91] Zhou Z.’e., Tang Y., Jin X. (2016). Metformin inhibits advanced glycation end products-induced inflammatory response in murine macrophages partly through AMPK activation and RAGE/NF*κ*B pathway suppression. *Journal of Diabetes Research*.

[92] Proks P., Reimann F., Green N., Gribble F., Ashcroft F. (2002). Sulfonylurea stimulation of insulin secretion. *Diabetes*.

[93] Kanazawa K., Kudo A. (2005). Self-assembled RANK induces osteoclastogenesis ligand-independently. *Journal of Bone and Mineral Research*.

[94] Ma P., Gu B., Ma J. (2010). Glimepiride induces proliferation and differentiation of rat osteoblasts via the PI3-kinase/Akt pathway. *Metabolism*.

[95] Fronczek-Sokol J., Pytlik M. (2014). Effect of glimepiride on the skeletal system of ovariectomized and non-ovariectomized rats. *Pharmacological Reports*.

[96] Yamada C., Yamada Y., Tsukiyama K. (2008). The murine glucagon-like peptide-1 receptor is essential for control of bone resorption. *Endocrinology*.

[97] Henriksen D. B., Alexandersen P., Hartmann B. (2007). Disassociation of bone resorption and formation by GLP-2: a 14-day study in healthy postmenopausal women. *Bone*.

[98] Xie D., Zhong Q., Ding K. H. (2007). Glucose-dependent insulinotropic peptide-overexpressing transgenic mice have increased bone mass. *Bone*.

[99] Schwartz A. V., Sellmeyer D. E., Ensrud K. E. (2001). Older women with diabetes have an increased risk of fracture: a prospective study. *The Journal of Clinical Endocrinology and Metabolism*.

[100] Ivers R. Q., Cumming R. G., Mitchell P., Peduto A. J., Blue Mountains Eye Study (2001). Diabetes and risk of fracture: the Blue Mountains Eye Study. *Diabetes Care*.

[101] Nicodemus K. K., Folsom A. R., Iowa Women's Health Study (2001). Type 1 and type 2 diabetes and incident hip fractures in postmenopausal women. *Diabetes Care*.

[102] Cornish J., Callon K. E., Bava U., Kamona S. A., Cooper G. J. S., Reid I. R. (2001). Effects of calcitonin, amylin, and calcitonin gene-related peptide on osteoclast development. *Bone*.

[103] Taylor S. I., Blau J. E., Rother K. I. (2015). Possible adverse effects of SGLT2 inhibitors on bone. *The Lancet Diabetes and Endocrinology*.

[104] Bilezikian J. P., Watts N. B., Usiskin K. (2016). Evaluation of bone mineral density and bone biomarkers in patients with type 2 diabetes treated with canagliflozin. *The Journal of Clinical Endocrinology and Metabolism*.

[105] Bays H. E., Weinstein R., Law G., Canovatchel W. (2014). Canagliflozin: effects in overweight and obese subjects without diabetes mellitus. *Obesity*.

[106] Ways K., Johnson M. D., Mamidi R. N. V. S., Proctor J., de Jonghe S., Louden C. (2014). Successful integration of nonclinical and clinical findings in interpreting the clinical relevance of rodent neoplasia with a new chemical entity. *Toxicologic Pathology*.

[107] CDC National diabetes statistics report. Estimates of diabetes and its burden in the United States. https://www.cdc.gov/diabetes/pdfs/data/statistics/national-diabetes-statistics-report.pdf.

